# Structural Connectivity of Functionally Defined Episodic Memory Networks: A Large‐Scale Connectome–Behavior Study

**DOI:** 10.1002/brb3.71515

**Published:** 2026-05-30

**Authors:** Neda Ghasemi, David Coynel, Andreas Papassotiropoulos, Dominique J. F. de Quervain

**Affiliations:** ^1^ Division of Cognitive Neuroscience, Department of Biomedicine University of Basel Basel Switzerland; ^2^ Research Cluster Molecular and Cognitive Neurosciences University of Basel Basel Switzerland; ^3^ Division of Molecular Neuroscience, Department of Biomedicine University of Basel Basel Switzerland; ^4^ University Psychiatric Clinics University of Basel Basel Switzerland

**Keywords:** brain networks, diffusion MRI, episodic memory, structural connectivity

## Abstract

**Background:**

Previous task‐based fMRI work in a large cohort of young adults identified nine large‐scale functional brain networks whose encoding‐related responsivity was robustly associated with individual differences in episodic memory. It remains unclear whether structural connectivity within these task‐fMRI‐defined networks explains inter‐individual variability in memory performance.

**Methods:**

We analyzed diffusion MRI data from 1,156 young adults who completed a picture‐free‐recall task. For each participant, we reconstructed whole‐brain diffusion MRI tractography and extracted within‐network connectivity for nine independent components previously defined by task‐based fMRI. Edge‐wise multiple regression models related within‐network connectivity strength to free recall performance, controlling for age and sex. False discovery rate (FDR) correction was applied across all tested edges and networks.

**Results:**

No associations between within‐network structural connectivity and memory performance survived FDR correction. Several edges showed nominal (uncorrected) associations with memory, corresponding to very small effect sizes (maximum *r* < 0.1).

**Conclusions:**

In contrast to previous findings for functional responsivity during encoding, within‐network structural connectivity in these functionally defined episodic memory networks is only weakly related to individual differences in memory in young healthy adults.

## Graphical abstract

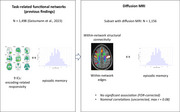

## 1 Introduction

Episodic memory—the ability to remember specific past events, along with their temporal and spatial context—is fundamental to adaptive behavior. The critical role of the medial temporal lobe in episodic memory was discovered in early lesion studies (Scoville and Milner 1957). Other studies have also identified regions, including the prefrontal and parietal cortices, the bilateral fusiform gyrus, and the occipital cortex, as being involved in episodic memory (Freedberg 2023; Hall 2024; Hutchinson et al. 2009; Staresina and Davachi 2008). More recent studies have shown that large‐scale brain networks appear to support episodic memory (Barnett et al. 2021; Fjell et al. 2016; Matyi and Spielberg 2022). Task‐based fMRI studies have revealed brain regions and networks associated with inter‐individual differences in episodic memory (Geissmann et al. 2023). Moreover, resting‐state studies have shown that intrinsic connectivity within medial temporal and default‐mode subnetworks, as well as between the hippocampus and prefrontal/parietal control regions, is related to individual variability in episodic memory performance, particularly in aging samples (Alm et al. 2022; Snytte et al. 2024; Van Buuren et al. 2019).

The brain's structural connectome may also contribute to individual differences in episodic memory. At the whole‐brain connectome level, picture free recall in healthy young adults was associated with global structural connectivity and with specific nodal and interregional connectivity patterns, including temporal, occipito‐temporal, insular, hippocampal, and subcortical connections (Coynel et al. 2017). Similarly, structural graph‐theoretical analyses showed that episodic memory performance was related to node strength within regions of the posterior medial memory system, particularly the parahippocampal cortex (Matyi and Spielberg 2022). Tract‐specific studies provide complementary evidence. In healthy older adults, age‐related recall decline was most closely associated with fornix microstructure, whereas uncinate fasciculus microstructure was linked to performance in a visual object–location associative memory task (Metzler‐Baddeley et al. 2011). Episodic source memory in older adults was further associated with white‐matter injury in the uncinate and inferior longitudinal fasciculi, with additional involvement of thalamo‐frontal projections, the superior longitudinal fasciculus, and the dorsal cingulum bundle (Lockhart et al. 2012). More recently, fornix and hippocampal cingulum microstructure, together with salience‐network functional connectivity, were shown to independently predict delayed episodic memory in older adults (Alm et al. 2022).

Currently, the reliability of brain behavior relationships is a major challenge. Evidence from large‐scale studies indicates that brain‐behavior relationships have, at best, small to medium effect sizes and only become stable when sample sizes reach the thousands of participants (Marek et al. 2022). In line with this, a recent study demonstrated weak and unstable prediction of behavioral traits such as personality from the structural connectome, especially in small or moderately sized samples (Rauland et al. 2025). These findings, together, emphasize the need for further investigations of brain‐behavior relationships in large samples.

Functional network organization is shaped, in part, by the underlying white‐matter architecture, although the relationship between structural and functional connectivity is not one‐to‐one (Honey et al. 2009; Mišić et al. 2016). Moreover, regional structure–function coupling — the degree to which functional connectivity profiles align with structural connectivity profiles — varies across individuals and has been associated with cognitive measures (Gu et al. 2021). These findings provide a rationale for testing whether individual differences in anatomical connectivity within task‐relevant functional networks are related to variability in memory performance.

In a recent task‐related functional network‐based brain–behavior correlation analysis in 1,498 young adults, we found that individual differences in visual episodic memory performance were related to network responsivity during encoding of nine functional networks identified through independent component analysis (Geissmann et al. 2023). Building on this prior work, the present study tested whether structural connectivity within these same task‐fMRI‐defined networks explains individual differences in free recall performance. This analysis was intended to test one specific form of structure–function convergence, rather than a direct replication of the task‐fMRI findings, because structure–function coupling is only partial and varies across cortical systems (Horn et al. 2014; Vázquez‐Rodríguez et al. 2019). Unlike previous studies focusing on global structural‐connectome metrics, predefined anatomical tracts, or resting‐state networks, our analysis directly examined whether anatomical connectivity within functionally defined memory‐related networks accounts for inter‐individual variability in episodic memory. To this end, we analyzed a subset of participants from Geissmann et al. (2023) for whom diffusion MRI was available (n = 1,156). Using tractography, we quantified structural connectivity within the nine networks and examined its association with free recall performance while adjusting for age and sex.

## 2 Materials and Methods

### 2.1 Participants

Data presented in this paper were part of a single‐center, large‐scale study of healthy young adults collected from 2008 to 2015 (Geissmann et al. 2023). From the total population of 1832 subjects, 449 had no diffusion data, and 143 were excluded because of missing T1 data or T1 data that did not pass visual inspection (e.g., excessive movement or scanner noise). An additional 74 subjects’ connectome reconstruction failed, and a further 10 subjects were excluded because of missing memory scores. Consequently, we analyzed data from 1,156 participants (724 females, 432 males; aged 18–35). Of the 1,498 participants reported by Geissmann et al. (2023), approximately 77.2% were also included in the current sample of 1,156 participants. At the time of the experiment, the subjects had no neurological or psychiatric conditions and were not taking any medication, except for hormonal contraceptives. Before participating, all subjects provided written informed consent. The ethics committee of the Canton of Basel, Switzerland, approved the study protocol. After a brief introduction and training session on the picture encoding task, subjects were guided into the MRI scanner to perform one run of the picture encoding task, followed by a separate working memory task, while fMRI data were being collected. Approximately 20 min later, an unannounced free recall task outside the scanner was performed. Subjects returned to the scanner for a second run, which included the acquisition of anatomical and diffusion images. This paper will focus on the free recall task, combined with anatomical and diffusion MRI data.

### 2.2 Episodic Memory Assessment

Episodic memory was evaluated using a picture‐free recall task. Participants first viewed 72 images (24 negative, 24 neutral, and 24 positive) along with 24 scrambled images presented in the MRI scanner through MR‐compatible LCD goggles (VisualSystem, NordicNeuroLab). Emotional and neutral pictures were selected from the International Affective Picture System (Lang et al. 2008) based on normative valence ratings. Among the 24 neutral pictures, eight were selected from an in‐house set to match the emotional pictures for visual complexity and content (e.g., human presence). The scrambled pictures contained 24 distinct, simple geometrical figures (rectangles or ellipses of different sizes and orientations), overlaid on a scrambled background composed of the IAPS pictures positioned one next to another, edited with a distortion and crystal filter in such a way that the motives were no longer perceivable. Pictures were displayed for 2.5 s in a quasi‐randomized, event‐related design with a 500 ms fixation cross and 9–12 s jittered inter‐trial intervals. Participants used a three‐point rating system to assess the valence and arousal of each picture during these intervals. For the scrambled pictures, the size and shape of the geometrical figures were judged. An unannounced paper‐pencil free recall test was given outside the scanner about 20 min after scanning, during which participants briefly described the pictures they remembered. Primacy and recency items, as well as training images, were excluded. The total number of correctly recalled pictures was used as a measure of episodic memory, and recall performance was evaluated by two independent raters (inter‐rater reliability > 98%). These data have been used in prior studies, and parts of the methods section were adapted from those papers (Coynel et al. 2017; Geissmann et al. 2023; Spalek et al. 2023).

### 2.3 MRI Acquisition

#### 2.3.1 MRI Scanner Parameters

The MRI scans were acquired on a Siemens Magnetom Verio 3T whole‐body scanner. It had a 12‐channel head coil. The high‐resolution structural scan was done using the magnetization‐prepared gradient echo (MPRAGE) protocol. The imaging parameters were TR = 2000 ms, TE = 3.37 ms, TI = 1000 ms, flip angle = 8°, 176 slices along the sagittal plane, FOV = 256 mm, and voxel resolution 1 × 1 × 1mm^3^. Diffusion‐weighted imaging was performed using single‐shot EPI with 64 diffusion‐weighted volumes (b = 900s/mm^2^), and one non‐diffusion‐weighted image (b = 0s/mm^2^). The diffusion parameters were TR = 9000 ms, TE = 82 ms, 58 slices along the axial plane, FOV = 320 mm, GRAPPA acceleration factor of 2, voxel resolution 2.5 × 2.5 × 2.5mm^3^.

### 2.4 MRI Preprocessing

All preprocessing steps were performed using QSIPrep 0.19.1, built on Nipype 1.8.6 (Gorgolewski et al. 2011). Many internal operations of QSIPrep use Nilearn 0.10.2 (RRID: SCR_001362) (Abraham et al. 2014) and Dipy 1.5.0 (Garyfallidis et al. 2014).

#### 2.4.1 Anatomical Data Preprocessing

The T1‐weighted image was corrected for intensity non‐uniformity (INU) using N4BiasFieldCorrection (Tustison et al. 2010) and ANTs 2.4.3 and used as an anatomical reference throughout the workflow. The anatomical reference image was reoriented into AC‐PC alignment via a 6‐DOF transform extracted from a full affine registration to the MNI152NLin2009cAsym template. A full nonlinear registration to the template from AC‐PC space was estimated via symmetric nonlinear registration (SyN) using antsRegistration. Brain extraction was performed on the T1w image using SynthStrip (Hoopes et al. 2022), and automated segmentation was performed using SynthSeg (Billot et al. 2023) from FreeSurfer version 7.3.1.

#### 2.4.2 Diffusion Data Preprocessing

Denoising was performed using MP‐PCA (Veraart et al. 2016) with a 5‐voxel window. B1 field inhomogeneities were corrected using the N4 algorithm via MRtrix3's dwibiascorrect after the corrected images were resampled. Motion and eddy current distortions were corrected using FSL's eddy (v6.0.5.1; (Andersson and Sotiropoulos 2016), with five iterations, a q‐space smoothing factor of 10, and 1000 voxels used to estimate hyperparameters. A linear first‐level model and a linear second‐level model were used to characterize eddy current‐related spatial distortion. q‐space coordinates were assigned to shells. Eddy‐current‐induced distortions and subject motion were estimated and corrected using FSL's eddy. Shells were aligned post‐eddy. Eddy's outlier replacement was run (Andersson et al. 2016). Data were grouped by slice, only including values from slices determined to contain at least 250 intracerebral voxels. Groups deviating by more than 4 standard deviations from the prediction had their data replaced with imputed values. Final interpolation was performed using the Jacobian method, and the preprocessed diffusion data were resampled to 1 mm isotropic resolution in AC‐PC‐aligned space.

#### 2.4.3 Whole‐Brain Tractography

QSIPrep‐preprocessed T1w images and brain masks were used to reconstruct subject‐specific whole‐brain fiber orientations, following QSIrecon's *mrtrix_singleshell_ss3t_ACT‐hsvs* workflow. A hybrid surface/volume segmentation was created. FreeSurfer outputs were registered to the QSIPrep outputs. Multi‐tissue fiber response functions were estimated using the DHollander algorithm from MRtrix3. Fiber orientation distributions (FODs) were assessed via constrained spherical deconvolution (CSD) (Tournier et al. 2004, Tournier et al. 2008) using an unsupervised multi‐tissue method (Dhollander, Thijs 2019; Dhollander et al. 2016). A single‐shell‐optimized multi‐tissue CSD was performed using MRtrix3Tissue (https://3Tissue.github.io), a fork of MRtrix3 (Tournier et al. 2019). FODs were intensity‐normalized using mtnormalize (Raffelt et al. 2017). Whole‐brain tractography was performed using MRtrix3's tckgen command, which uses the iFOD2 probabilistic tracking method to generate 1e7 streamlines with a maximum length of 250 mm, a minimum length of 30 mm, and an FOD power of 0.33. T1w segmentation was used for anatomical constraints, whereby streamlines are seeded at the white matter–gray matter interface and terminated upon entering non‐brain tissue.

#### 2.4.4 Networks and Nodes

**FIGURE 1:**
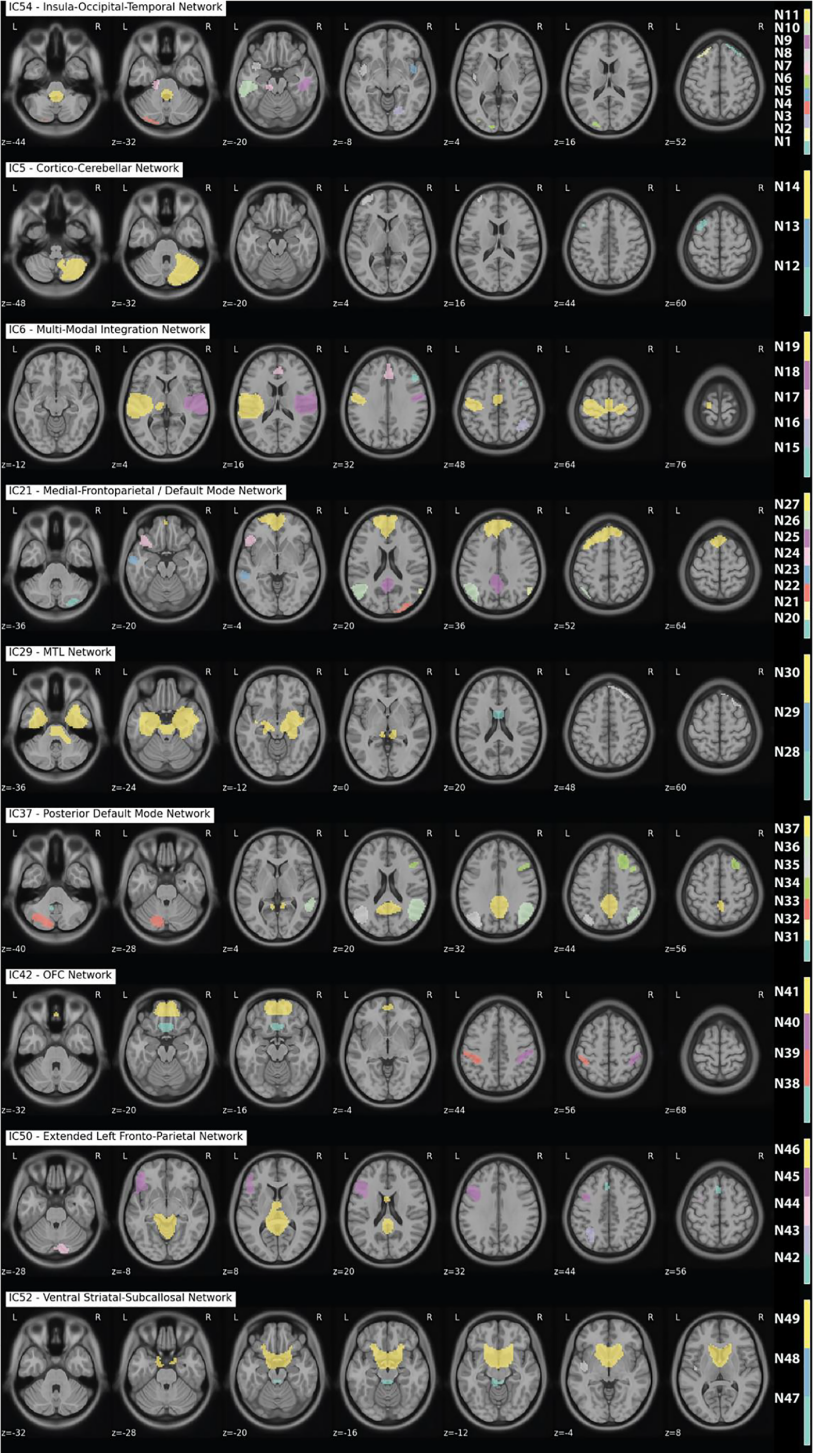
Functional network clusters, extracted from the 9 independent components for which the functional responsivity during picture encoding exhibited a significant association with inter‐individual episodic memory performance in Geissmann et al. (2023). Colors denote distinct nodes within each IC and are for visualization only.

Our analyses focused on the within‐network structural connectivity of previously identified functional networks from the encoding task data (Geissmann et al. 2023). The functional networks were extracted using independent component analysis (ICA), which identified 60 independent components (ICs). In particular, we were interested in the 9 networks for which the functional responsivity during picture encoding exhibited a significant association with individual episodic memory performance. A network of interconnected brain regions is represented in each IC. IC 5 (Cortico‐Cerebellar Network), which includes the right cerebellum and left fronto‐opercular, fronto‐caudal, fronto‐rostral, temporal, and parietal areas. IC‐21 (Medial‐Frontoparietal / Default Mode Network) contains the frontal pole, medial OFC, superior frontal cortex, rostral ACC, PCC, precuneus, isthmus cingulate, occipital cortices, and angular gyrus. IC‐29 (Medial Temporal Lobe Network) comprises the parahippocampal gyrus, hippocampus, entorhinal cortex, amygdala, brainstem, thalamus, and right cerebellum. IC‐37 (Posterior Default Mode Network) involves the precuneus, posterior cingulate, intracalcarine and lingual gyri, pre‐ and postcentral gyri, angular gyrus, middle temporal gyrus, supramarginal gyrus, lateral occipital cortex, and parts of the left cerebellum. The medial OFC and bilateral postcentral gyrus are in the IC‐42 (Orbitofrontal Cortex Network). The superior frontal cortex, opercular cortex, lateral OFC, rostral and caudal frontal cortex, inferior frontal cortex, cerebellum, precuneus, PCC, brainstem, thalamus, and angular gyrus are included in the IC‐50 (Extended Left Frontoparietal Network). The nucleus accumbens, caudate, subcallosal cortex, and OFC are contained in the IC‐52 (Ventral Striatal‐Subcallosal Network). IC‐54 (Insula‐Occipital‐Temporal Network) includes occipital, temporal, hippocampal, insular, precentral, subcallosal, brainstem, and ventricular regions and partially overlaps with the other ICs. IC‐6 (Multi‐Modal Integration Network) overlays sensory‐motor and sensory‐auditory areas, including anterior and posterior cingulate cortices and the posterior insula. Responsivity of IC 6 demonstrated a negative association with the number of pictures freely recalled, while the other significant ICs showed a positive association. Between 3 and 11 contributing clusters were identified per IC by considering the voxels with the highest contribution within each network (z‐value greater than 3) and a cluster size of at least 50 voxels. The resulting functional network clusters are illustrated in Figure 1. Because approximately 77.2% of the participants in this study originated from the cohort of Geissmann et al. (2023), we transformed the network clusters from functional group‐level MNI space back into the subject‐specific QSIPrep AC‐PC‐aligned space. This was done by applying a series of transformations from MNI to individual T1w space (combining MNI‐to‐DARTEL template affine transformation and DARTEL template‐to‐T1w flow field), followed by a rigid transformation from T1w space to AC‐PC‐aligned space (computed through antsRegistrationSyNQuick.sh).

#### 2.4.5 Within‐Network Connectivity

The whole‐brain tractograms were used to extract within‐network connectivity for each of the functional networks of interest, using MRtrix3's tck2connectome command. Within‐network connectivity was stored as adjacency matrices with edge weights defined by the number of reconstructed streamlines (Gruber et al. 2023). To account for variations in streamline count across subjects, matrices were normalized by the total number of valid streamlines generated.

#### 2.4.6 Connectivity–Behavior Analysis

**FIGURE 2:**
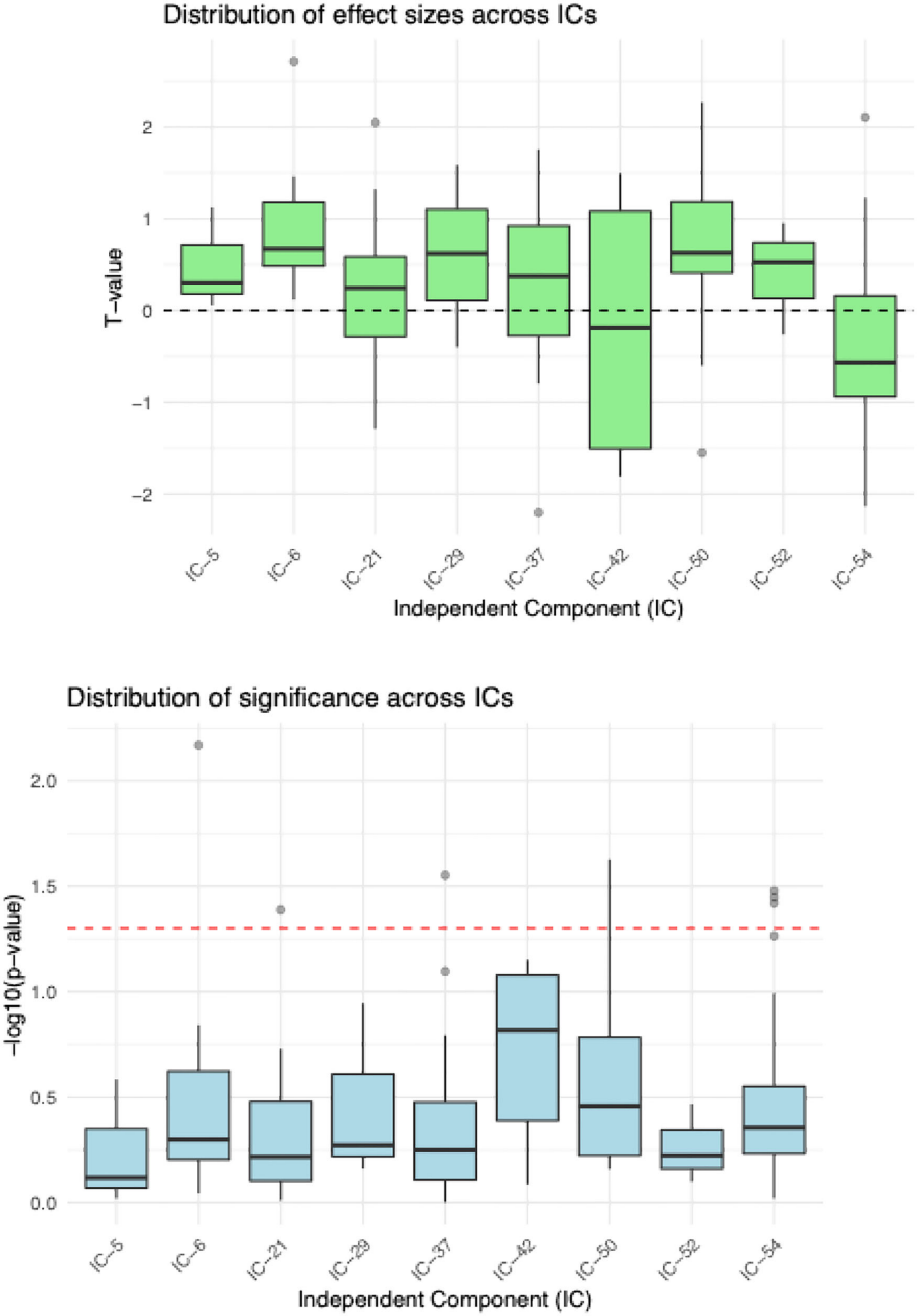
Associations between structural connectivity and episodic memory performance. Upper panel: Boxplots showing the distribution of t‐values for regression coefficients linking edge‐wise structural connectivity to memory performance within each independent component (IC). Lower panel: Boxplots of the corresponding uncorrected −log10(p) values across edges. In both panels, each box summarizes all tested edges within the respective IC. Horizontal lines indicate medians, boxes represent interquartile ranges, whiskers reflect variability across edges, and individual points indicate edge‐wise associations outside the whisker range. The red dashed line in the lower panel indicates nominal significance at *p* < 0.05, uncorrected. No association survived correction for multiple comparisons.IC‐5: Cortico‐Cerebellar Network; IC‐6: Multi‐Modal Integration Network; IC‐21: Medial Frontoparietal / Default Mode Network; IC‐29: Medial Temporal Lobe Network; IC‐37: Posterior Default Mode Network; IC‐42: Orbitofrontal Cortex Network; IC‐50: Extended Left Frontoparietal Network; IC‐52: Ventral Striatal–Subcallosal Network; IC‐54: Insula–Occipital–Temporal Network.

The relationship between structural connectivity and episodic memory performance was investigated using multiple linear regression to link edge weights within each independent component (IC) to free recall scores while controlling for age and sex. We calculated the t‐statistic and associated p‐values for each edge. For visualization, Figure 2 summarizes the distribution of edge‐wise regression t‐values and corresponding uncorrected −log10(p) values across all tested edges within each independent component.

Multiple comparisons increased the risk of Type I error because multiple regression models were run across all edges within each IC. Therefore, Benjamini and Hochberg's false discovery rate (FDR) correction method (correcting across all edges and ICs) was applied to the raw p‐values derived from the model. Statistical significance was defined as an FDR‐adjusted p‐value threshold of less than 0.05.

## 3 Results

### 3.1 Associations Between Within‐IC Connectivity and Memory Performance

Consistent with the sample reported by Geissmann et al. (2023), free‐recall performance showed substantial inter‐individual variability in the present diffusion MRI subset. The number of pictures freely recalled ranged from 5 to 55 (M  =  30.90, SD  =  8.29; see also (Geissmann et al. 2023). From functional network‐based analyses, the strength of within‐network connectivity was extracted for nine independent components (ICs). Memory performance was used as the dependent variable in multiple regression models for each IC, controlling for sex and age. No association remained after multiple comparison correction (FDR, *q* < 0.05). Nonetheless, several ICs showed nominally significant associations (uncorrected *p* < 0.05). Figure 2 summarizes the distribution of edge‐wise regression t‐values across ICs in the upper panel and the corresponding uncorrected −log10(p) values in the lower panel. The most nominally significant results were obtained by IC‐6 (multi‐modal integration network; *t* = 2.71, *p* = 0.0067), IC‐50 (extended left frontoparietal network; *t* = 2.26, *p* = 0.023), and IC‐37 (posterior default mode network; *t* = ‒2.19, *p* = 0.028). IC‐54 (insula–occipital–temporal network) contributed three additional edges with nominal significance (*t* = ‐2.13–2.10, *p* = 0.033–0.039), while IC‐21 (medial frontoparietal / default mode network) showed a weaker effect (*t* = 2.04, *p* = 0.040).

The following regions were involved in those edges:

The right caudal anterior cingulate cortex and right rostral anterior cingulate cortex were involved in N17, and N18 included the right superior temporal cortex and adjacent right cerebral white matter, all within IC‐6 (multi‐modal integration network). The right cerebellar cortex and right cerebellum white matter were included in N44, and the left isthmus cingulate cortex and left cerebellum cortex were included in N46, related to IC‐50 (extended left frontoparietal network). Related to IC‐21 (medial frontoparietal / default mode network), the left middle temporal cortex was included in N23 and adjacent white matter, while the left isthmus cingulate cortex and left precuneus were included in N25. IC‐37 (posterior default mode network): The right caudal middle frontal cortex was contained in N34, and adjacent white matter was contained in N34; the left inferior parietal cortex and adjacent white matter were contained in N35. IC‐54 (insula–occipital–temporal network): N3 with the right lingual cortex and adjacent white matter and N7 with the left cerebellum cortex and left cerebellum white matter; N6 including the left lateral occipital cortex and adjacent white matter and N8 comprising the left superior temporal cortex and left insula; N1 with the right superior frontal cortex and right rostral middle frontal cortex and N11 spanning the right and left cerebellum cortex.

## 4 Discussion

In this study, which included 1,156 participants, we investigated whether structural connectivity within nine functionally defined brain networks explains inter‐individual differences in episodic memory performance. In our previous work, we identified specific functional connectivity patterns within these nine network components whose responsivity during encoding was robustly associated with episodic memory performance (Geissmann et al. 2023). In contrast, the present analysis revealed that within‐network structural connectivity in these networks was not significantly associated with memory performance at the given sample size: Although several nominal associations emerged, none survived correction for multiple comparisons. Given the large sample size, this null finding is informative and suggests that within‐network streamline‐based structural connectivity of these task‐fMRI‐defined networks does not explain a substantial proportion of inter‐individual variability in episodic memory in healthy young adults. This interpretation is further supported by the very small observed effect sizes, with the largest nominal association explaining less than 1% of the variance.

Of the nine IC networks, five networks showed nominally significant associations with memory performance. Because these effects are small and did not survive correction, the following observations regarding network anatomy should be interpreted as exploratory. Associations were located in the IC 6 (multi‐modal integration network) and IC 21 (medial frontoparietal / default mode network), including the anterior cingulate, superior temporal gyrus, precuneus, and isthmus cingulate areas, which are implicated in emotion and memory, sensory information processing, and language (Rolls 2019; Sasaki et al. 2023). IC 50 (extended left frontoparietal) consists of the cerebellum along with limbic regions (isthmus cingulate), known to be involved in episodic memory and emotional processes (Kollenburg et al. 2024; Melka et al. 2024). Regions of IC 37 (posterior default mode network), representing the posterior default mode network including the inferior parietal and caudal middle frontal regions, showed correlation with working‐memory performance (Østby et al. 2011). Among the large number of regions of IC 54 (insula–occipital–temporal), the lingual gyrus (in the middle occipital cortex) plays a role in emotion as well as visual and non‐visual functions such as verbal memory (Palejwala et al. 2021). The cerebellum also contributes to working‐memory performance (Ding et al. 2012). Additionally, the insula is known for a broad range of functions, including emotion processing (Snytte et al. 2024), and reduced connectivity in the left temporo‐occipital cortex, where increased communicability was observed, has been associated with worse memory performance (Llufriu et al. 2019). Based on the largest nominal effect (IC 6, *t* = 2.71, *r* ≈ 0.08), power calculations (two‐sided) indicate that ∼3,000 would be needed under stringent correction across the 139 tested edges (power 80%, Bonferroni α = 0.05/139) for such associations to survive multiple‐comparison control; even then, they would explain <1% of the variance in episodic memory.

Taken together with our previous functional findings (Geissmann et al. 2023), these results suggest that inter‐individual variability in episodic memory in healthy young adults is more strongly related to dynamic encoding‐related network responsivity rather than to stable white‐matter connectivity within these networks.

## 5 Limitations

First, structural connectivity was estimated from single‐shell diffusion MRI using CSD‐derived FOD‐based probabilistic tractography, and connectivity strength was quantified using streamline counts. Although this approach allows whole‐brain structural connectivity to be reconstructed, streamline counts are indirect computational estimates and should not be interpreted as direct measures of axonal count, white‐matter integrity, myelination, or communication efficiency. They can be influenced by methodological factors such as seeding strategy, region size, tract length, crossing fibers, fiber geometry, and bottleneck effects, which may lead to false‐positive or false‐negative connections (Donahue et al. 2016; Schilling et al. 2020; Smith et al. 2022; Sotiropoulos and Zalesky 2019). More advanced acquisitions and models, such as multi‐shell diffusion and higher‐order reconstruction methods, may provide a more accurate characterization of white‐matter architecture and detect subtler associations with memory performance. Second, it is unclear whether these results generalize to older adults or clinical populations, because the sample consisted of young, healthy adults. Third, our analysis focused on within‐network structural connectivity of task‐fMRI‐defined ICA networks. Such ICA components identify distributed regions with shared task‐related BOLD responsivity, but they do not necessarily correspond to anatomically defined tracts or structural modules. Moreover, structure–function relationships are not one‐to‐one and vary across regions and individuals (Gu et al. 2021; Horn et al. 2014; Mišić et al. 2016; Vázquez‐Rodríguez et al. 2019). The absence of robust associations therefore does not rule out a role of white‐matter architecture in episodic memory. Memory‐related anatomical effects may be better captured by other features, such as tract‐specific temporal or medial‐temporal pathways, between‐network structural connections, or broader graph‐theoretical properties of the structural connectome (Berlot et al. 2016; Matyi and Spielberg 2022).

## 6 Conclusion

The present study investigated whether structural connectivity within nine functional networks previously linked to individual differences in episodic memory contributes to the observed inter‐individual variability in memory performance. Although no association survived correction for multiple comparisons, some edges showed nominal (uncorrected) relationships with memory. These findings suggest that, in young healthy adults, within‐network structural connectivity in these functionally defined networks explains at most a small proportion of the variance in episodic memory performance.

## Author Contributions

**Neda Ghasemi**: writing – original draft, writing – review and editing, formal analysis, visualization, investigation, validation, methodology. **Andreas Papassotiropoulos**: writing – review and editing, funding acquisition, supervision, project administration, conceptualization. **David Coynel**: visualization, writing – original draft, writing – review and editing, formal analysis, data curation, supervision, methodology, validation. **Dominique J.F. de Quervain**: project administration, conceptualization, funding acquisition, writing – review and editing, supervision.

## Funding

This work was supported by a Swiss Government Excellence Scholarship awarded to NG and institutional funding from the Division of Cognitive Neuroscience, University of Basel.

## Conflicts of Interest

The authors declare no conflicts of interest.

## Data Availability

The data supporting the findings of this study are available on the Open Science Framework (OSF): https://doi.org/10.17605/OSF.IO/MN45H.
